# Dimethyl disulfide exerts antifungal activity against *Sclerotinia minor* by damaging its membrane and induces systemic resistance in host plants

**DOI:** 10.1038/s41598-020-63382-0

**Published:** 2020-04-16

**Authors:** Swati Tyagi, Kui-Jae Lee, Pratyoosh Shukla, Jong-Chan Chae

**Affiliations:** 1 Division of Biotechnology, Jeonbuk National University, Iksan, 54596 Republic of Korea; 2 Enzyme Technology and Protein Bioinformatics Laboratory, Department of Microbiology, Maharshi Dayanand University, Rohtak, 124001 Haryana India

**Keywords:** Microbiology, Applied microbiology

## Abstract

Microbial volatile compounds (MVCs) significantly influence the growth of plants and phytopathogens. However, the practical application of MVCs at the field level is limited by the fact that the concentrations at which these compounds antagonize the pathogens are often toxic for the plants. In this study, we investigated the effect of dimethyl disulfide (DMDS), one of the MVCs produced by microorganisms, on the fitness of tomato plants and its fungicidal potential against a fungal phytopathogen, *Sclerotinia minor*. DMDS showed strong fungicidal and plant growth promoting activities with regard to the inhibition of mycelial growth, sclerotia formation, and germination, and reduction of disease symptoms in tomato plants infected with *S. minor*. DMDS exposure significantly upregulated the expression of genes related to growth and defense against the pathogen in tomato. Especially, the overexpression of *PR1* and *PR5* suggested the involvement of the salicylic acid pathway in the induction of systemic resistance. Several morphological and ultrastructural changes were observed in the cell membrane of *S. minor* and the expression of ergosterol biosynthesis gene was significantly downregulated, suggesting that DMDS damaged the membrane, thereby affecting the growth and pathogenicity of the fungus. In conclusion, the tripartite interaction studies among pathogenic fungus, DMDS, and tomato revealed that DMDS played roles in antagonizing pathogen as well as improving the growth and disease resistance of tomato. Our findings provide new insights into the potential of volatile DMDS as an effective tool against sclerotial rot disease.

## Introduction

In nature, plants are exposed to a wide range of phytopathogens that cause severe yield loss and increase the farming cost as a result of the expenses incurred in controlling them^1^. Fungi belonging to the genera *Sclerotinia*, *Alternaria*, *Fusarium*, *Rhizoctonia*, *Botrytis* and *Phytophthora* are common phytopathogens with a broad host range and can survive under unfavorable conditions by forming special structures, such as sclerotia^2^. Synthetic fungicides are used extensively to control the pathogens owing to their low costs and effective results^3^. However, the use of these compounds is detrimental to the living system, posing environmental safety issues, and causes the emergence of resistant pathogenic strains^4^. Many studies have been performed to develop methods for the biological control of plant pathogens either by application of antagonistic microorganisms or their secondary metabolites as an alternative to conventional pesticides^5–8^. Microbial volatile compounds (MVCs) have been verified to inhibit the growth of pathogens and to improve the health of plants by inducing a systemic resistance^4,6^. For example, *Bacillus* and *Pseudomonas* strains were reported to produce 2,3-butanediol, acetoin, 2-butanone, 2-methyl-n-1-tridecene, albuterol, 1,3 propane-di-ol, and dimethyl disulfide (DMDS), which improved the growth of plants by interfering with plant hormone signaling and induced systemic resistance against plant pathogens^6,8–11^. Recently, MVCs, mainly consisting of benzyl alcohol, propanol, butanol, and other sulfur-containing compounds, were reported to improve the growth of *Arabidopsis, Nicotiana*, and agaves^8,12^. The root system of *Arabidopsis* was modulated upon exposure to the volatile DMDS, which affected the canonical auxin signaling pathway^13^. Increased number of lateral roots and root hairs helped the plants to absorb more nutrients resulting in the improvement of their health. Similarly, the expression of several genes involved in the promotion of plant growth was reported to be upregulated when *Arabidopsis* seedlings were exposed to 2,3-butanediol produced by *Bacillus*^14–16^, and simultaneously their resistance against *Setosphaeria turcica*, *Spodoptera littoralis*, *Erwinia carotovora*, and the hemibiotrophic fungus, *Colletotrichum orbiculare*, was enhanced^17–19^. In addition to bacteria, MVCs from eukaryotes such as *Candida*, *Trichoderma*, *Hypocrea* were also reported to suppress the growth of aflatoxin producing fungi *Aspergillus flavus*^20^ and *Rhizoctonia solani*^21^.

Several studies have shown the potential of MVCs as alternatives to chemical pesticides and fertilizers^9,22^. However, an understanding of the mechanisms underlying the effects of these compounds and studies on their application *in situ* has been limited although there are a few reports for action mode such as DNA damage by *N*-methyl-*N*-nitrosoisobutyramide generated from fungi^23,24^. In nature, MVCs are produced as mixtures of several compounds in which the composition and concentration of each compound is not well defined and differs in the producing microorganisms, depending on the nutrient availability and metabolic activity^9,22,25,26^. Although MVCs can act as multitrophic signals in ecologically complex systems and can elicit pleiotropic responses^25,26^, the practical application of MVCs as alternatives to chemical pesticides requires more information, such as accurate identification and assessment of the bioactive compounds.

In this study, we used exogenous application of DMDS, which is produced by several bacteria, including *Pseudomonas*, *Serratia*, *Bacillus*, and *Stenotrophomonas*, as the most abundant compound in their MVC mixtures, to control the growth of a wide range of plant pathogens^18,26,27^. Although several studies have indicated the antimicrobial potential of DMDS^28–30^, nothing related to its mode of action is known. Herein, we evaluated the antagonistic activity of exogenous DMDS application against fungal plant pathogens, specifically *Sclerotinia minor*, and the promotion of growth and induction of systemic resistance in DMDS-treated plants by assessing the expression of genes involved in growth and disease resistance in tomato.

## Results

### Minimum inhibitory concentration (MIC) and antifungal activity of DMDS against fungal phytopathogens

*In vitro*, exposure to volatile DMDS inhibited the growth of all the tested fungal phytopathogens (Table 1). The MICs of DMDS for *Aspergillus flavus* and *Rhizoctonia solani* were higher than 50 µM, whereas for other fungi, the values were 50 µM or less (Table 1). Treatment with volatile DMDS also resulted in phenotypic changes in fungi, such as loss of pigments and morphological alterations in the hyphae (Table 1). The growth of DMDS-treated fungi was restored when they were re-inoculated on fresh medium but the abnormalities were still seen in the hyphae. Therefore, DMDS-mediated damage of fungal mycelium appeared to be fungistatic.

**Table 1:** Effect of DMDS on different plant pathogenic fungi.

Fungal strains	Species	Plant host	Disease	MIC (µM)	Percent inhibition	Phenotypic characteristics			
						Aerial hyphae	Growth retardation	Pigmentation	Sporulation
*Sclerotinia minor*	KACC41068	Tomato	Sclerotinia rot	50	81.1 ± 13.9	−	+	ND	ND
*Sclerotinia sclerotium*	KACC40172	Citrus	White mold	50	83.1 ± 10.8	−	+	ND	ND
*Fusarium graminarum*	KACC41040	Barley	Scab	50	89.5 ± 11.3	ND	+	−	ND
*Fusarium oxysporum*	KACC40037	Tomato	Fusarium wilt	50	84.8 ± 11.0	ND	+a	−	ND
*Aspergillus niger*	KACC47429	Onion	Black mould	50	81.3 ± 06.9	ND	ND	−	−
*Aspergillus flavus*	KACC41809	peanut	Aspergillus rot	100	82.7 ± 12.2	ND	+	−	ND
*Penicillium digitatum*	KACC42258	Citrus	Green rot	25	93.5 ± 05.2	−	+	ND	ND
*Fusarium oxysporum*	KACC48266	Reddish	Fusarium wilt	25	96.3 ± 04.5	ND	+^a^	−	ND
*Rhizectonia solani*	KACC40123	Ginseng	Damping off	75	80.5 ± 09.7	ND	+	ND	ND

^a^Slow growth; ND, not determined.

**Figure 1:**
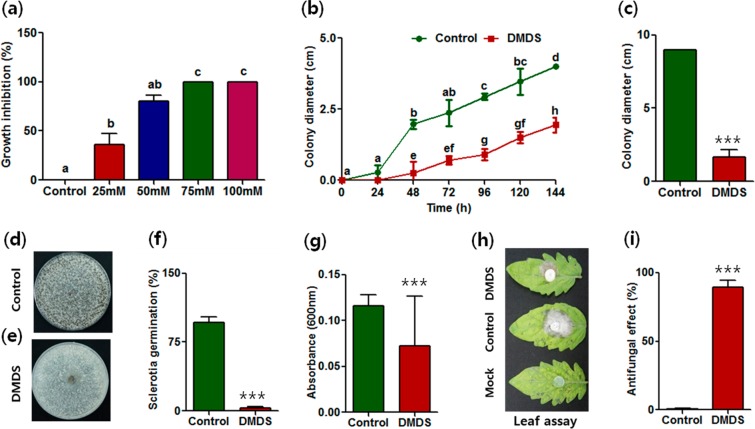
Antifungal activity of volatile dimethyl disulfide (DMDS) on *Sclerotinia minor*. (**a**) Inhibition of fungal growth at different concentrations of DMDS, (**b**) antifungal activity of DMDS up to 144 h, (**c**) antifungal activity of DMDS in the dual-plate assay, (**d**,**e**) sclerotia formation, (**f**) sclerotia germination, (**g**) mycelium attachment, **(h**) leaf detachment assay showing the effect of DMDS on disease development, (**i**) percentage antifungal effect determined in detached leaf assay. Results are the mean values for five replicates. Small letters above the error bars represent significant differences according to the Bonferroni’s multiple comparison test (*p* value = 0.05). Asterisks indicate significant changes in the values calculated by Student’s *t*-test (***p < 0.001).

*S. minor*, a sclerotia-forming pathogen with a wide host range, was selected for further studies. To investigate the antagonistic effect of DMDS on fungal mycelia, sclerotia formation, and germination, dual-plate assays were performed. At 50 µM, DMDS inhibited the mycelial growth in *S. minor* and at concentrations between 75 and 100 µM it caused complete inhibition of the fungal growth (Fig. 1a). The growth of *S. minor* was reduced up to 70.42% within three days of exposure to volatile DMDS (50 µM) in comparison to that in control-2 (see Fig. 1b, Supplementary Fig. 1a, and “Methods” section for the control sets). No significant differences in growth were observed in control-1, 2, and 3 (Supplementary Fig. 1a). On the other hand, volatile DMDS was adsorbed by activated charcoal in control-4 and its antifungal effect on the pathogen was not detected (Supplementary Fig. 1a). Hence, control-2 was used as the control for further studies. Under control conditions, the fungal mycelia showed the maximum growth whereas DMDS exposure reduced the mycelial growth arising from the fungal plug (Fig. 1c). A growth reduction of 81.34% was observed after five days of DMDS treatment (Supplementary Fig. 1b). A similar effect of volatile DMDS on sclerotia formation (the ability of fungi to produce new sclerotia) and germination (the ability of already formed sclerotia to geminate and produce new hyphae) was observed. A significant amount of healthy and viable sclerotia was formed under the control conditions (Fig. 1d) whereas very few non-viable sclerotia, with abnormal shape and size, were generated upon treatment with DMDS (Fig. 1e), indicating the antifungal effect of volatile DMDS. Furthermore, DMDS treatment also inhibited the germination of sclerotia by up to 99% when compared to the germination in the control (Fig. 1f).

Mycelial attachment on the surface of hosts is the primary event in disease progression^30^. DMDS exposure might affect the mycelial attachment, restricting the penetration of the pathogen and its colonization of the host, and thereby reducing the disease incidence. Therefore, mycelial attachment and disease index were determined after the DMDS treatment. The mycelial attachment was reduced by 51% after DMDS treatment (Fig. 1g). The disease index after DMDS treatment, as determined by detached leaf assay, was reduced by 70% compared to that in the control (Fig. 1h), with an overall antifungal effect of 89% (Fig. 1i).

### Growth promotion of tomato plants

**Figure 2:**
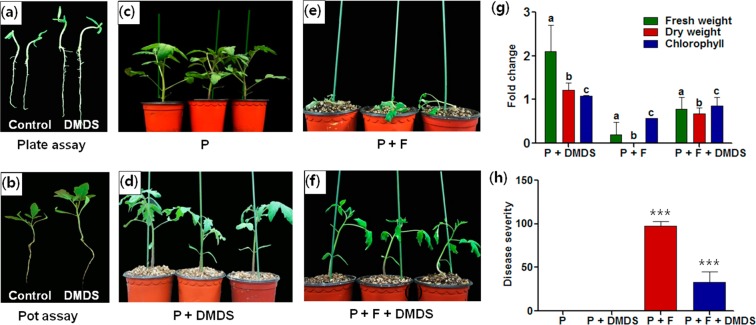
Effect of volatile dimethyl disulfide (DMDS) on the growth of tomato in the plate and pot assay. (**a**) Control and DMDS-treated plants in the plate assay, **(b**) plants in the pot assay, (**c**) non-treated plant, (**d**) plants treated with DMDS, (**e**) plants infected with *Sclerotinia minor*, (**f**) *S. minor* infected plants under DMDS treatment, (**g**) fold change in fresh, dry and chlorophyll content depending on the experimental conditions, (**h**) disease severity. P, plant; F, fungi. Results are the mean values for five replicates. Small letters above the error bars represent significant differences according to Bonferroni’s multiple comparison test (*p* value = 0.05). Asterisks indicate significant changes in the values calculated by Student’s *t*-test (***p < 0.001).

To prevent the growth of *S. minor*, 50 µM of DMDS was sufficient under the experimental conditions used in this study. The effect of this concentration of DMDS on the growth of tomato plants was assessed using plants grown in airtight tissue culture dishes and pots. Upon DMDS treatment, plants grown in plates as well as in pots exhibited a significant increase in fresh weight, dry weight, root length, shoot length, leaf area, and chlorophyll content when compared to the control (Fig. 2a,b, Supplementary Fig. 2). When grown in plates, the effect on the shoot length was most significant, with an increase of 70% compared to that in the control. The fresh and dry weights were increased by about 55% and 50% and the leaf area and chlorophyll content were increased by about 50% and 15%, respectively. Similarly, a significant improvement in the growth was also observed in the pot assay, indicating the potential of DMDS in an open system. In the pot assay, the effect was more prominent on the dry weight and leaf area (which increased by 62% and 48%, respectively), whereas the shoot length was increased only by 30% (Fig. 2b, Supplementary Fig. 2).

### Biocontrol of *S. minor* infection in tomato plants

The antifungal efficacy of volatile DMDS against *S. minor* infection in tomato was investigated. Under non-treated and DMDS-treated conditions, no disease symptoms were observed in the plants. The growth of DMDS-treated plants was significantly improved compared to that of the non-treated control plants (Fig. 2c,d). In the non-treated plants, infection with *S. minor* resulted in severe disease symptoms after 7 days. The mycelial growth was observed on stems and roots. The leaves showed necrosis and chlorosis, the crown tissue was destroyed, and eventually the plants perished (Fig. 2e). Plants infected with *S. minor* showed less disease symptoms (about 70% less) when they were treated with DMDS (Fig. 2f). Plants in all the three treatments showed a noticeable difference in weight (fresh and dry) and chlorophyll content compared to the non-treated controls (Fig. 2g). Exposure to DMDS alone increased the fresh weight, dry weight, and chlorophyll content of the plants by 2-, 1-, and 0.9-fold, respectively. In the case of co-treatment with the pathogen and DMDS, all the parameters were improved almost by 1.2-fold when compared with the respective parameters in the plants infected with *S. minor*. DMDS treatment antagonized the pathogen and was able to improve the plant growth. Notably, symptoms of the sclerotial rot disease were reduced significantly in plants treated with DMDS (Fig. 2h).

### Hydrogen peroxide (H_2_O_2_) accumulation and callose deposition

**Figure 3:**
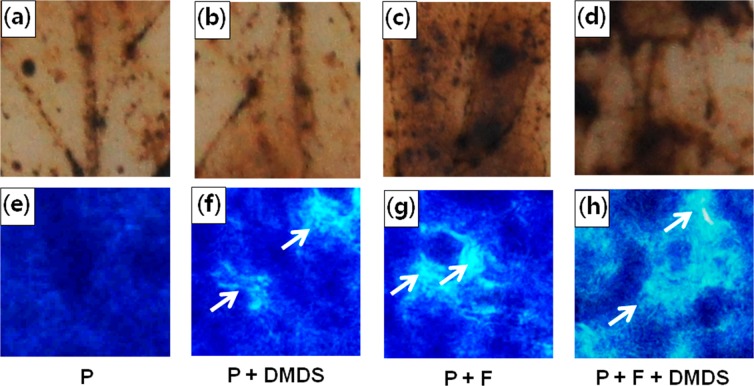
Accumulation of hydrogen peroxide and callose. H_2_O_2_ accumulation in (**a**) healthy plants, (**b)** dimethyl disulfide (DMDS)-treated plant, (**c**) *Sclerotinia minor* infected plants, and (**d**) *S. minor* infected plants under DMDS treatment; callose deposition in (**e**) healthy plants, (**f**) DMDS-treated plants, (**g**) *S. minor* infected plants, and (**h**) *S. minor* infected plants under DMDS treatment.

Infection with a necrotrophic fungus causes oxidative stress, resulting in the increase in reactive oxygen species (ROS) levels in plants, which further accelerates the infection by enhancing the fungal growth as well as necrosis in plants^31^. On the other hand, callose deposition has known to protect plants from invasion of plant pathogens^32^. Therefore, we determined the production of H_2_O_2_ and deposition of callose in tomato plants in response to *S. minor* infection by 3,3′-diaminobenzidine (DAB) and methyl blue staining, respectively. The H_2_O_2_ level was increased in plants infected with *S. minor*, indicating the increase in oxidative stress (Fig. 3c). However, non-treated and DMDS-treated plants showed no (Fig. 3a,b) or less (Fig. 3d) H_2_O_2_ accumulation. Furthermore, deposition of callose was noticeably enhanced with DMDS treatment, indicating the role of DMDS in host defense (Fig. 3e–h). Taken together, these results suggest that DMDS enhances the cellular defense responses.

### Transcriptional analysis of the effects of DMDS on tomato

**Figure 4:**
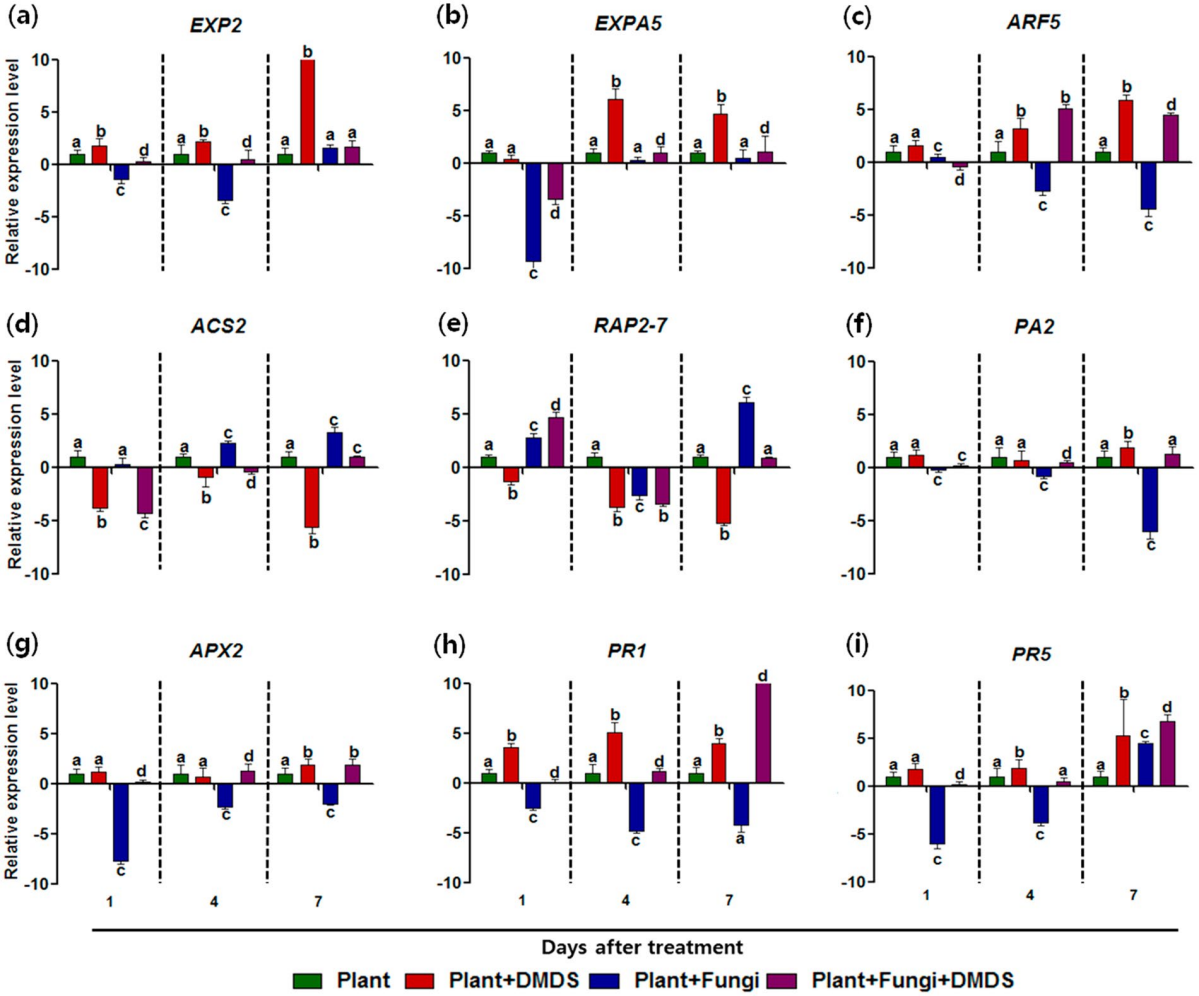
Transcriptional profiles of genes involved in plant growth regulation and disease resistance after exposure to volatile dimethyl disulfide (DMDS). Transcriptional profiles of (**a**) *EXP2*, (**b**) *EXPA5*, (**c**) *ARF5*, (**d**) *ACS2*, (**e**) *RAP2-7*, (**f**) *PA2*, (**g**) *APX2*, (**h**) *PR1*, and (**i**) *PR5*. Error bars indicate standard deviation. Small letters above the error bars represent significant differences according to Bonferroni’s multiple comparison test (*p* value = 0.05).

Growth promotion and resistance in plants are related to the expression of phytohormones and pathogenicity-related^15,16^. To investigate if DMDS influenced the expression of genes involved in pathogenicity or in phytohormone synthesis, the transcriptional levels of genes for expansin (*EXP2* and *EXPA5*) and auxin response factor (*ARF5*), and of those involved in ethylene production (*ACS2* and *RAP2-7*), oxidation (*APX2* and *PA2*), and pathogenicity related (*PR1* and *PR5*) were examined using quantitative real-time PCR (qRT-PCR). As shown in Fig. 4, DMDS treatment significantly altered the relative expression of all these genes. The expression of *EXP2* and *EXPA5* were upregulated after DMDS treatment compared to their expression in the control. However, reduced expression of these genes with respect to control was detected in the pathogen-infected plants. DMDS treatment of pathogen-infected plants caused an upregulation of both *EXP2* and *EXPA5* over that in the non-treated plants (Fig. 4a,b). A noticeable reduction in the expression of *ARF5* (involved in auxin synthesis) was observed in pathogen-infected plants compared to that in the control. However, DMDS treatment induced an upregulation of *ARF5* expression both in the presence and absence of the pathogen (Fig. 4c). The expression of *ACS2* and *RAP2-7* was downregulated after DMDS treatment both in uninfected and pathogen-infected plants whereas their expression was upregulated in the untreated plants infected with *S. minor* (Fig. 4d,e). The peroxidase genes, *APX2* and *PA2*, were significantly upregulated after DMDS treatment. However, pathogen infection resulted in the downregulation of *APX2* and *PA2* (Fig. 4f,g). Noticeably, *PR1* and *PR5* were also upregulated after treatment with volatile DMDS. The pathogen-infected plants showed a significant decrease in the expression of both these genes (Fig. 4h,i).

### Morphological changes in *S. minor*

To investigate the mode of action of DMDS, morphological and ultrastructural studies were performed. Because cell wall and cell membrane are the primary targets of several fungicides^33^, we examined the integrity of cell wall and cell membrane by measuring the electrolyte leakage in DMDS-treated or untreated hyphae after incubation for 4 days. Almost 49.2% of electrolyte leakage was observed in the treatment condition as compared to 8.2% in the control (Supplementary Fig. 3).

**Figure 5:**
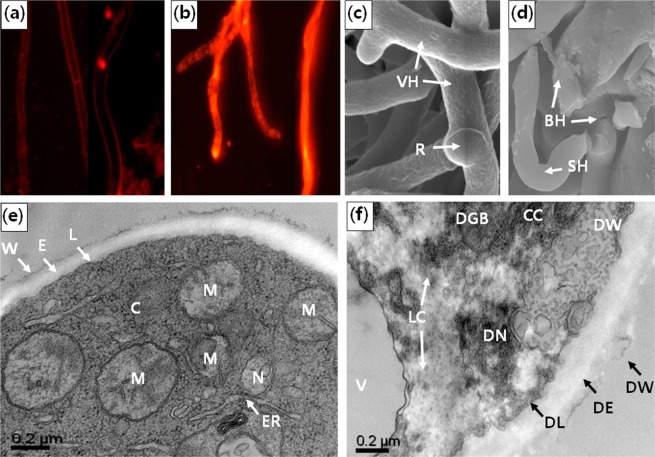
Morphological changes caused by dimethyl disulfide (DMDS) in *Sclerotinia minor* Fluorescence micrograph of *S. minor* under (**a**) control and (**b**) treated conditions. Hyphae were stained with propidium iodide (PI) at a concentration of 10 µL/mL. (**c**) Scanning electron micrograph showing ultra-structures of *S. minor* hyphae in under control conditions. (**d**) Scanning electron micrograph showing ultrastructural changes in *S. minor* when mycelia were exposed to volatile DMDS. (**e**) Transmission electron micrograph showing the ultrastructure of *S. minor* hyphae under control conditions. (**f**) Transmission electron micrograph showing ultrastructural changes in *S. minor* when mycelia were exposed to volatile DMDS. VH = vegetative hyphae, R = rind, BH = broken hyphae, SH = swollen hyphae, C = cytoplasm, M = mitochondria, W = wall, E = extracellular matrix, L = electron dense line, ER = Endoplasmic reticulum, N = Nucleus, V = vacuole, CC = condensed cytoplasm, LC = lost cytoplasm, DW = deformed wall, SE = swollen extracellular matrix, DL = degenerated electron line, DGB = degenerated Golgi body, DN = distorted nucleus.

The integrity of cell wall and cell membrane was investigated by fluorescence, scanning, and transmission electron microscopy. First, the DMDS-treated hyphae were stained with propidium iodide (PI) and observed under a fluorescence microscope. In the cells with disturbed membrane integrity, PI is internalized and produces red fluorescence whereas it cannot cross the membrane of healthy cells, making it useful to check the membrane integrity. The penetration of PI in the control and DMDS-treated cells was visualized and is shown in Fig. 5a,b. In the control, hyphae were not stained with PI whereas DMDS-treated hyphae were stained and appeared red. This indicated that DMDS affected the cell wall integrity of the pathogen. Furthermore, morphological and ultrastructural abnormalities in the DMDS-treated hyphae were observed by scanning electron microscopy and transmission electron microscopy. In the control, the hyphae were healthy, uniform, and linear and did not show any abnormalities (Fig. 5c) whereas DMDS-treated hyphae were abnormal, swollen, broken, and degenerated in appearance (Fig. 5d). Uniform cell wall/membrane, normal cytoplasm, organelles, vacuoles, regular-shaped endoplasmic reticulum, Golgi bodies, and mitochondria were seen in the control hyphae (Fig. 5e). However, DMDS-treated samples exhibited significant changes like detachment and loosening of cell membrane from the cell wall, disrupted cell wall, cytoplasmic condensation, loss of cytoplasm, degenerated organelles, larger vacuoles, and accumulated cytoplasmic materials (could be lipids or proteins) (Fig. 5f).

### Ergosterol content and demethylase gene expression

**Figure 6:**
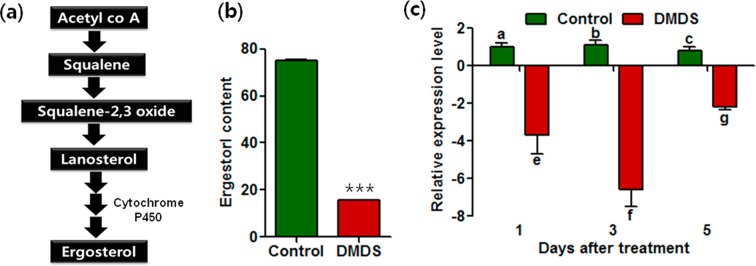
Analysis of ergosterol biosynthesis in dimethyl disulfide (DMDS)-treated and non-treated fungi. (**a**) Biosynthetic pathway of ergosterol, (**b**) ergosterol content in the control and DMDS-treated fungi, and (**c**) relative expression of *CYP51* at different time intervals. Error bars indicate standard deviation. Small letters above the error bars represent significant differences according to the Bonferroni’s multiple comparison test (*p* value = 0.05). Asterisk indicates a significant change in the values calculated by Student’s *t*-test (***p < 0.001).

Ergosterol is the main sterol in fungal membranes that contributes to cell wall/membrane integrity, fluidity, and cell metabolism^34^. Its biosynthesis is mainly controlled by a cytochrome P450, lanosterol 14α-demethylase gene (*CYP51*)^35^ (Fig. 6a). This gene is one of the primary targets of several known fungicides. Sulfur-containing compounds were also reported to interfere with the oxidative phosphorylation in eukaryotes^30^. Hence, the effect of DMDS on ergosterol synthesis in *S. minor* was investigated by high performance liquid chromatography. After DMDS treatment, the ergosterol content was reduced by up to 80% compared to that in the control (Fig. 6b; Supplementary Fig. 4). In addition, the transcription of *CYP51* was significantly downregulated by the DMDS treatment (Fig. 6c). The expression of *CYP51* was reduced up to 3.7-, 6.6-, and 2.1-fold after 1, 3, and 5 days of DMDS treatment, respectively. Considered along with the observations on the cell structure, these results suggest the inhibition of ergosterol biosynthesis as the main mode of action through which DMDS influenced the growth of *S. minor*.

## Discussion

Volatile organic compounds of microbial origin have been reported to promote plant growth and to play antagonistic roles against plant pathogens^9,22^. However, few studies have been conducted on the mode of action of such compounds. In a previous study, we observed a dose-dependent effect of DMDS on the growth of *Arabidopsis* and its interference with the auxin signaling pathways^13^. In this study, we show that volatile DMDS not only controls the fungal pathogens but can also induce resistance against *S. minor* in tomato. *S. minor* is a devastating fungal pathogen with a broad host range and produces sclerotia that can survive unfavorable conditions for years^30^. These sclerotia can germinate again under favorable conditions and initiate infection of host plants^30^. DMDS inhibited the growth of *S. minor* as well as of other pathogenic fungi and also caused structural changes, such as loss of pigmentation and distorted hyphae (Table 1). These structural alterations might play a significant role in the reduction of virulence and pathogenicity of the pathogens^36^. The exposure to DMDS not only arrested the mycelial growth (87%) of *S. minor* but also had a significant effect on the formation and germination of sclerotia. DMDS treatment completely repressed the formation and germination of sclerotia.

The mode of action through which DMDS interferes with the growth of *S. minor* is still unclear. Several studies have reported that VOCs damage the cell membrane, causing the movement of intracellular material out of the cell and sometimes result in cell death^23,30,37^. In our study, DMDS treatment increased the electrolyte leakage by about 49.2% compared to that in the untreated control which is consistent with the previous study where isoliquiritin affected the growth of *Peronophythora litchi* Chen by damaging the plasma membrane of the pathogen and increased the electrolyte leakage by 50%^38^. Also, eugenol and other compounds, such as 2-methyl butanol and 3-methyl butanol, were reported to increase the concentration of potassium ions and cellular materials in fungi, and the antifungal activity was due to the alteration of membrane permeability and cell wall disruption^37–39^. Further, PI staining and results of scanning and transmission electron microscopy clearly showed the differences in the integrity of cell wall and cell membrane in the DMDS-treated and non-treated hyphae (Fig. 5). The results indicated that the disruption of cell wall and membrane integrity and altered permeability affected the mycelial growth and pathogenicity, resulting in abnormal fungal growth or cell death^37,38^. A similar phenomenon of altered morphology and spore germination was reported in plant pathogens, *Botrytis cinerea*, *Penicillium italicum*, and *Sclerotinia sclerotium*, when they were exposed to microbial VOCs^30,37–39^. Our results indicated that DMDS controlled the pathogen growth through a mechanism involving membrane damage.

Volatile DMDS promoted plant growth and induced resistance in tomato. The tomato seedlings cultivated *in vivo* and *in vitro* displayed improved plant growth, assessed in terms of fresh and dry weight, root and shoot length, and chlorophyll content (Fig. 2). The results are in agreement with previous studies in which acetoin, 2,3-BD, and DMDS produced by *Bacillus*, *Pseudomonas*, *Stenotrophomonas*, and *Burkholderia* improved plant growth by increasing the chlorophyll content, altering phytohormone expression, and providing the sulfur nutrient under sulfur-limiting conditions^10,13–16,27^.

Disease development and progression in plants require physical attachment of a pathogen with its host^30^. Attachment of mycelium to the host is necessary for a fungus to invade the host plant properly and to colonize^30^. Interestingly, our results revealed that DMDS treatment reduced the attachment ability of mycelia by almost 51%, which in turn affected the host colonization by 70% in detached leaf assay and improved the antifungal effect up to 89% (Fig. 1). The pathogenicity of necrotrophic pathogens, such as *S. minor*, is directly related to the superoxide and hydrogen peroxide levels^40^. In this study, infection of tomato plants with *S. minor* led to the accumulation of H_2_O_2_ in the plants along with development of disease whereas the infected plants that were treated with DMDS accumulated lesser amount of H_2_O_2_ (Fig. 3). At the molecular level, the expression of *APX2* and *PA2*, the peroxidases that detoxify ROS, was upregulated when *S. minor* infected plants were exposed to DMDS. Moreover, deposition of callose (β-1,3-glucan polymer) is known to be an important mechanism in the response to (a)biotic stresses through which hosts interrupt the colonization and multiplication of pathogens^32^. Enhanced callose deposits in powdery mildew resistant *Arabidopsis* were observed at the fungal penetration site suggesting that callose deposition was responsible for penetration resistance to powdery mildew^41^. *Bacillus cereus* AR156 exhibiting suppressive activity to *B. cinerea* also induced more accumulation of H_2_O_2_ and callose in *Arabidopsis*^42^. Defensive role of callose synthase was suggested in *Citrus* responding to *Liberibacter asiaticus* through the increase of callose deposition which resulted in reducing bacterial colonization^43^. In this study, callose deposition was also observed in *S. minor* infected and non-infected plants. However, the infected plants showed maximum deposition of callose when they were treated with DMDS (Fig. 3). These results suggested that DMDS exposure activated the plant defense system and provided resistance against the pathogen infection.

Under pot conditions, plants infected with *S. minor* showed severe disease symptoms. However, disease development was reduced in the infected plants upon DMDS treatment. The reduction in the disease development in DMDS-treated plants was related to the antifungal effects of DMDS, which include the ability to reduce the colonization of pathogen and to induce the expression of growth and defense-related genes in plants (Fig. 4). The genes for expansin, auxin, and ethylene biosynthesis are responsible for plant growth promotion whereas peroxidase and PR genes contribute to disease resistance^15–17^. In *Arabidopsis*, the transcription of genes responsible for the homeostasis, signaling, and transport of auxin, ethylene, and expansin was reported to be modulated by mixtures of MVCs produced by *Bacillus* and *Pseudomonas*, resulting in the growth promotion and enhanced disease resistance of plants^15–18^. In this study, tomato exhibited the transcriptional upregulation of genes for expansin (*EXP2, EXPA5*) and auxin (*ARF5*) when exposed to DMDS whereas these genes were downregulated in the presence of *S. minor*. Expansins are known to play a role in the loosening of plant cell walls; suppression of expansins enhanced the resistance of *Arabidopsis* to the necrotrophic fungus, *Alternaria brassicicola*^44^. Interestingly, the expression of *EXP* was decreased when *S. minor* infected plants were treated with DMDS compared to its expression in plants that were only treated with DMDS. The transcriptional down-regulation of *EXP* might assist plants in resisting the pathogen infection. The expression and action of expansins under biotic stress is complicated and is influenced by other phytohormones, such as abscisic acid, auxins, brassinosteroids, cytokinins, and ethylene^27,45^. Therefore, in this study, the expression of *EXP* might have been influenced by enhanced levels of auxin and ethylene responsive genes in *S. minor* infected plants under DMDS treatment although it remains unclear as to how these hormones interacted with each other.

The expression of *ARF5* was also upregulated when the plants infected with *S. minor* were treated with DMDS. Likewise, exogenous application of auxin was reported to increase plant growth and induce systemic resistance against *Fusarium*, indicating its role in disease resistance^46^.

The ethylene biosynthesis genes (*ACS2* and *RAP2-7*) were downregulated after DMDS treatment suggesting the involvement of DMDS in the promotion of plant growth. The overproduction of ethylene gene in plants results in dwarfed phenotype, with reduced growth patterns^15–17,27^. The ethylene response pathway is also related to defense against necrotrophic pathogens^47^ and upregulation of *ACS2* and *RAP2-7* genes was detected in *S. minor* infected plants compared to the healthy plants. Plants exert different types of defense response depending on the infecting pathogens^48^. For biotrophs, salicylic acid (SA)-mediated defense system is activated whereas jasmonic acid or ethylene dependent systems are triggered against necrotrophic pathogens^48^. *PR1* and *PR5* are activated by SA and most of the PR proteins are reported to inhibit fungal growth^49^. The expression of PR genes was upregulated in *Arabidopsis* responding to pathogens when exposed to volatile compounds^6,50^. The application of volatile 3-pentanol primed the transcriptional level of PR genes in cucumber and pepper and activated the SA defense pathway^6,51^. MVCs emitted from *B. subtilis* and *Paenibacillus polymyxa* also improved the expression of *PR* genes which induced immune response against plant pathogens via SA pathway^52,53^. In this study, the transcriptional upregulation of *PR1* and *PR5* after DMDS treatment with or without pathogenic attack (Fig. 4h,i) suggested that DMDS accelerated the induction of systemic resistance mediated by SA signaling pathway in tomato because PRs are known to be the main components of the SA pathway^48,49^.

In conclusion, we investigated the tripartite interaction among a pathogenic fungus, DMDS, and tomato, which resulted in the suppression of sclerotial rot in this study. DMDS did not only suppress the pathogenesis of *S. minor* by reducing the virulence through membrane damage mechanism, but also activated the plant growth promotion and induced systemic resistance against sclerotial rot in tomato. The Florida Department of Agriculture and Consumer Services (FDACS) have declared DMDS as a safe compound that does not have any negative impact on the humans and the environment^54^. Therefore, DMDS could be an antifungal compound with high potential as an alternative to pesticides.

## Methods

### Plant and fungal growth conditions

Tomato seeds (cultivar cerasiforme; Green Heart Bio Pvt. Ltd., South Korea) were surface sterilized using 0.1% mercury chloride, as described by Tyagi *et al*.^13^, and grown aseptically on sterilized filter paper. For *in planta* experiments, young seedlings were transferred to pots filled with sterilized soil (gardening soil:vermiculite:perlite = 4:3:3) and incubated in the plant-growth chamber under 16-h day/8-h night condition with 200 μmol/m^2^/sec light intensity at 24 °C. The fungal pathogens used in this study (Table 1) were obtained from the Korean Agricultural Culture Collection (KACC) center, and grown and maintained on potato dextrose agar (PDA) medium (MB cell, South Korea) at 25 °C. DMDS, ergosterol, methanol, and acetonitrile (>95%, HPLC grade) were purchased from Sigma–Aldrich (Germany).

### Analysis of *in vitro* antifungal activity

The ability of volatile DMDS to inhibit the fungal growth was assessed using the I plate technique^55^. The DMDS stock solution was prepared in ethanol and further diluted to solutions in the concentration range of 25–100 µM. Lanoline (1.6 g) solution prepared in dichloromethane (10 mL) was mixed with each dilution of DMDS solution in a 1:1 ratio and 50 μL of the solution was dropped onto a filter paper disc. The MIC was defined as the lowest concentration that caused an 80% decrease in fungal growth compared to the growth of untreated controls. While DMDS solution on a filter paper disc was placed in one compartment, fungal plugs were taken from the margins of a freshly grown colony with a sterile cork-borer (5-mm diameter) and placed on the PDA surface in the other compartment of the I plate. The plates were sealed with Parafilm M (Bemis, USA) to inhibit the leakage of the volatile compound. Fungal plates without DMDS were prepared as control. All the Petri dishes were incubated at 25 °C for 4 days. The diameter of the fungal colony, reduction in mycelial growth, and phenotypic characteristics were recorded after the incubation period. The reduction in the mycelial growth was calculated as follow^56^:

$${\rm{Mycelium}}\,{\rm{growth}}\,{\rm{reduction}}( \% )=[({\rm{Dc}}-{\rm{Dt}})/{\rm{Dc}}]\times 100$$

where Dc = diameter of the fungi in control, Dt = diameter of the fungi under the treatment condition

Further, the effects of volatile DMDS on the mycelial growth, sporulation (sclerotia formation), and sclerotia germination were investigated using the double-plate assay^56^. Briefly, a fresh agar plug of *S. minor* mycelia was inoculated into the center of a fresh PDA medium and a sterilized Durham’s tube filled with DMDS (50 μL volume of 50 μM) was attached on the other plate. Both the plates were inverted over each other, sealed with Parafilm-R (Bemis, USA) and incubated at 25 °C. Four different control sets were maintained: control-1 was prepared without DMDS and Parafilm sealing; control-2 was also without DMDS but the plates were sealed, considering the effect of the possibly preexisting VOCs produced by fungi; control-3 contained ethanol and was sealed to investigate the solvent effect on the fungal growth; control-4 was prepared with DMDS and activated charcoal (1 g) that was used to adsorb the gaseous DMDS. After every 24 h, the diameter of *S. minor* was recorded and the reduction in growth was calculated as discussed above. As described by Luo *et al*.^37^, detached leaf assay was performed to determine the attachment of mycelium, which reflected the hydrophobic surface tendency and fungal colonization in the host.

### Assay of plant growth promotion and antagonism of fungus

The plant growth promotion activity of DMDS was analyzed using plate and pot assays. For plate assay, 5-day-old tomato seedlings were exposed to DMDS in a dual-tissue culture plate and incubated vertically for a week^56^. Briefly, one of lidless petridishes containing MS agar medium and the others attached with a Durham’s tube for DMDS supply were laid in opposition, sealed together with Parafilm-R, and placed vertically in a plant growth chamber. For pot assay, tomato seedlings were grown as described by Tahir *et al*.^16^, with slight modifications. One-week-old tomato seedlings of equal size were transferred to soil-filled pots (8 cm × 6 cm) fitted over glass jars and wrapped with parafilm to prevent the leakage of the volatile compound. Each pot had small holes (10-mm diameter) in the bottom, allowing exposure of roots to the VOCs. Volatile DMDS was supplied in a sterilized Durham’s tube capped with cotton plug and attached to the wall of the jar. After incubation for 1 week at 28 °C, the plants were removed from the plates and pots, and different parameters, namely fresh and dry weight, root and shoot length, and chlorophyll content, were measured.

The effects of DMDS on the growth of tomato plants and on their resistance to the disease caused by *S. minor* were investigated. For the *in planta* experiment, the following four sets were made: (a) healthy plants, used as control, (b) plants treated with DMDS, (c) plants infected with *S. minor*, (d) plant treated with DMDS after infection with *S. minor*. All the plants were grown and maintained in pots as discussed above. The intact plant inoculation method was used for artificial infection of tomato with the pathogen, as previously described^57^. The main stem of the plant was scrapped with a razor blade and inoculated by placing the fungal agar plugs (2 plugs each plant) taken from the actively growing margin of fungal colony of *S. minor* with a sterile cork borer (5 mm diameter). The plants were treated with DMDS as discussed above and then kept in the plant growth chamber. At the end of the experiment, plant weight (fresh and dry), chlorophyll content, and disease severity were measured. Disease symptoms were recorded on a 0–4 disease severity scale; 0 = no disease symptom, 1 = 25% disease symptom, 2 = 50% disease symptom, 3 = 75% disease symptom, and 4 = 100% (death of the plant). The data for sclerotial rot were collected after 7 days of exposure to volatile DMDS, using the following formula^15^:

$${\rm{Disease}}\,{\rm{severity}}=\sum [({\rm{ni}}\times {\rm{vi}})/({\rm{N}}\times {\rm{V}})]\times 100$$

where ni is the number of plants with the respective disease rating, vi is disease rating, V is the maximum disease rating, and N is the total number of plants observed. The oxidative stress was determined in terms of hydrogen peroxide accumulation and callose deposition, as described by Nie *et al*.^42^.

### Transcriptional analysis

To evaluate the promotion of growth and disease resistance at the molecular level, the expression of growth- and defense-related genes was analyzed. Total RNA was extracted from the leaf samples collected on 1, 4, and 7 days of treatment using the Tri-RNA reagent (Ambion, USA) following the manufacturer’s instructions and purified using the RNeasy plant mini kit (Qiagen, USA). The first strand of cDNA was synthesized using the Primescript RT reagent kit (TaKaRa, Japan). Real-time PCR was performed with the StepOne RT-PCR system (Applied Biosystems, USA) using SYBR Green I (Enzynomics, South Korea). The relative expression levels of genes involved in expansin (*EXP2* and *EXPA5*), auxin (*ARF5*), and ethylene (*ACS2* and *RAP2-7*) biosynthesis, and those encoding peroxidase (*APX2* and *PA2*) and pathogenesis-related proteins (*PR-1* and *PR5*) were determined. The *ACT-2* (actin) gene was used as an internal reference. For qRT-PCR, the following program was used: 95 °C for 10 min, followed by 35 cycles of 95 °C for 30 s, 58 °C for 30 s, and 72 °C for 30 s. The sequences of the primers used are given in Supplementary Table 1. Three replicates were taken for each sample and the relative gene expression was estimated using the 2^−ΔΔCt^ method, as described by Livak & Schmittgen (2001)^58^.

In addition, real-time PCR was performed to analyze the transcriptional levels of *CYP51*, which is a key gene controlling ergosterol synthesis in fungi. The RNA samples were prepared from *S. minor* incubated in the presence or absence of DMDS for different time intervals (1, 3, and 5 days). RNA extraction and real-time PCR were performed as described above. ITS was used as an internal control and the PCR program was as follows: 95 °C for 10 min, followed by 35 cycles of 95 °C for 30 s, 55 °C for 30 s, and 72 °C for 30 s.

### Morphologically characterization

Electrolyte leakage was determined as described by Sharifi & Ryu (2016)^8^. The integrity of cell wall and plasma membrane of *S. minor* after exposure to DMDS was examined by fluorescence microscopy^38^. Four-day-old mycelia from PDA plates were stained with PI (10 μg/mL) in the dark for 30 min. Phosphate-buffered saline solution (pH 7.0) was used to remove the excess stain and then the samples were observed under the ECLIPSE Ti2 fluorescence microscope (Nikon, Japan). The external morphology and alterations in the ultrastructure of *S. minor* were examined using scanning and transmission electron microscopy after exposure to volatile DMDS for 4 days at 25 °C. For scanning electron microscopy, mycelia were first fixed in paraformaldehyde and 2% glutaraldehyde solution in 0.05 M sodium cacodylate buffer (pH 7.2). Secondary fixation was carried out with 1% osmium tetroxide in the same buffer (pH 7.2) at 4 °C for 2 h and then the samples were stained with 0.05% uranyl acetate. The samples were dehydrated by gradually increasing the concentration of ethanol, equilibrated with 100% isoamyl acetate, and then mounted for the observation. For thin sections, the dehydrated samples were embedded in a mixture of 100% propylene oxide and 812 resin. They were polymerized for 48 h. The sections were cut with an ultra-microtome and were stained with lead citrate before observation under a transmission electron microscope (H7650, Japan) at an accelerating voltage of 100 kV.

### Analysis of ergosterol

Ergosterol was extracted as described by Koch *et al*.^59^ and was quantified by high performance liquid chromatography^34^. In brief, the extracted samples were purified by filtering through a 0.22-µm syringe filter (Millipore, USA) and then injected (20 µL) into a C18 column (250 × 4.6 mm, 5-µm diameter, ACE, Scotland). The mobile phase was a mixture of methanol and acetonitrile (80:20, v/v) and was used at a flow rate of 0.6 mL/min. The effluent was monitored at 280 nm to detect ergosterol.

## Supplementary information

Supplementary Information.
