# Author Correction: Dimethyl disulfide exerts antifungal activity against *Sclerotinia minor* by damaging its membrane and induces systemic resistance in host plants

**DOI:** 10.1038/s41598-020-74901-4

**Published:** 2020-10-20

**Authors:** Swati Tyagi, Kui-Jae Lee, Pratyoosh Shukla, Jong-Chan Chae

**Affiliations:** 1 Division of Biotechnology, Jeonbuk National University, Iksan, 54596 Republic of Korea; 2 Enzyme Technology and Protein Bioinformatics Laboratory, Department of Microbiology, Maharshi Dayanand University, Rohtak, 124001 Haryana India

Correction to: *Scientific Reports* 10.1038/s41598-020-63382-0, published online 16 April 2020

This Article contains errors in the reference citations.

In the Results section under subheading ‘Minimum inhibitory concentration (MIC) and antifungal activity of DMDS against fungal phytopathogens’,

“Mycelial attachment on the surface of hosts is the primary event in disease progression^30^.”

should read:

“Mycelial attachment on the surface of hosts is the primary event in disease progression^8^.”

In the Discussion section,

“In our study, DMDS treatment increased the electrolyte leakage by about 49.2% compared to that in the untreated control which is consistent with the previous study where isoliquiritin affected the growth of *Peronophythora litchi* Chen by damaging the plasma membrane of the pathogen and increased the electrolyte leakage by 50%^38^.”

should read:

“In our study, DMDS treatment increased the electrolyte leakage by about 49.2% compared to that in the untreated control which is consistent with the previous study where isoliquiritin affected the growth of *Peronophythora litchi* Chen by damaging the plasma membrane of the pathogen and increased the electrolyte leakage by 50%^37^.”

Also in the Discussion section,

“Disease development and progression in plants require physical attachment of a pathogen with its host^30^. Attachment of mycelium to the host is necessary for a fungus to invade the host plant properly and to colonize^30^.”

should read:

“Disease development and progression in plants require physical attachment of a pathogen with its host^8^. Attachment of mycelium to the host is necessary for a fungus to invade the host plant properly and to colonize^8^.”

In the Methods section under subheading ‘Morphologically characterization’,

“Electrolyte leakage was determined as described by Sharifi & Ryu (2016)^8^.”

should read:

“Electrolyte leakage was determined as described by Luo et al (2016)^37^.”

In addition, this Article contains a typographical error in the Methods section subheading where,

“Morphologically characterization”

should read:

“Morphological characterization”

