# Supplementary material for: Dimethyl disulfide exerts antifungal activity against Sclerotinia minor by damaging its membrane and induces systemic resistance in host plants
Source: Sci Rep. 2020 Apr 16;10:6547. doi: 10.1038/s41598-020-63382-0 (PMC7162885; doi:10.1038/s41598-020-63382-0)

Supplementary information

Dimethyl disulfide exerts antifungal activity against *Sclerotinia minor* by damaging its membrane and induces systemic resistance in host plants

Swati Tyagi[1], Kui-Jae Lee[1], Pratyoosh Shukla[2*] & Jong-Chan Chae[1*]

[1]Division of Biotechnology, Jeonbuk National University, Iksan 54596, Republic of Korea.

[2]Enzyme Technology and Protein Bioinformatics Laboratory, Department of Microbiology, Maharshi Dayanand University, Rohtak 124001, Haryana, India.

*Corresponding authors

Supplementary Table 1. List of primers used in this study

Name	Primer sequence (5’⟶ 3’)	Target gene	Gene ID
ACT2-F	CCAGGCACACAGGTGTTATG	Actin	101267214
Act2-R	GCCTCAGTCAGGAGAACAGG		
EXP2-F	CGAACCCGTCTCTACCTAACA	Expansin	543582
EXP2-R	GGACGATACCGGCTTTGTATTT		
EXPA5-F	ACTTGAGCCTAAGCTTGTAGTC	Expansin	543558
EXPA5-R	CCGTATCCCGTGCTGTATAAAT		
ARF5-F	AGCTCCAATAGGCCAGGATT	Auxin response factor	100736448
ARF5-R	ACTGTGCTCCCGCTGTTACT		
ACS2-F	ATGTCGAGTTTCGGTTTAGTATCT	1-amino-cyclopropane-1-carboxylate synthase	606304
ACS2-R	CTAACCTCATCGCGCTTTCT		
RAP2-7-F	GTCACTCCAGTGCCAGTATTT	Ethylene-responsive transcription factor	101252772
RAP2-7-R	TAGTTGGTTGCTTGGTGGTAG,		
APX2-F	CAGGCTGTGACCACTTGAGA	Ascorbate peroxidase	778224
APX2-R	TCAAAACCAGAACGCTCCTT		
PA2-F	AGAAGAGCAAATCAAGGAGGAG	Peroxidase	101254854
PA2-R	AGTAATGGTGAGGCCAACAG		
PR1-F	AAAATGGTGGGCAAATTCAA	Pathogenesis-related protein	101267538
PR1-R	TTTTTCTCATCAGCCCAAGC		
PR5-F	GAGTCCTGGATTGCAAAGGA	Pathogenesis-related protein	101264959
PR5-R	AAGTGAACCAGGGCATTCAC		

Supplementary Figure 1. Effect of DMDS on fungal growth. (a) Dual plate assay showing the effect of DMDS on fungal growth, (b) fungal growth inhibition, (c) sclerotia formation. Asterisks indicate significant change in the values calculated by Student's *t*-test (***, p<0.001).

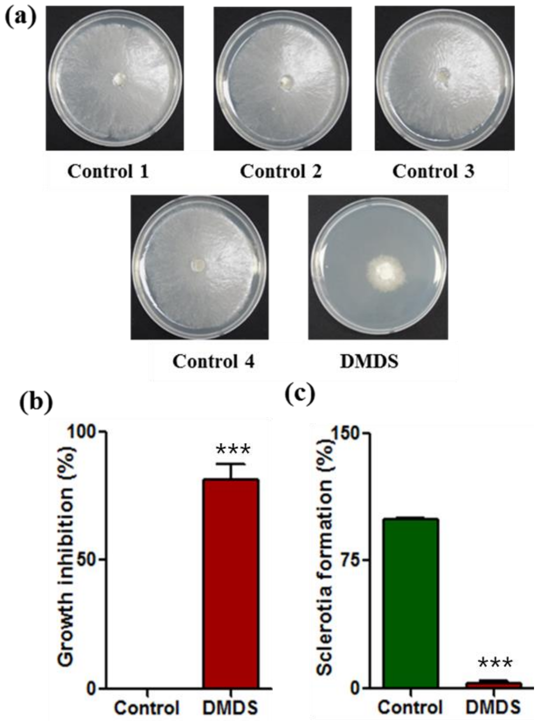

Supplementary Figure 2. Relative enhancement of different plant growth parameters in plate and pot assays. Small letters above the error bars represent significant differences according to the Bonferroni's multiple comparison test (p value = 0.05).

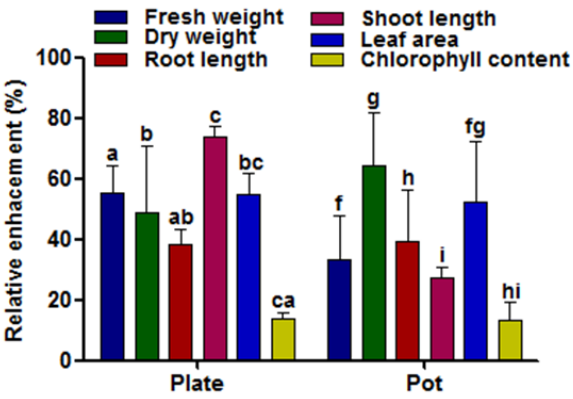

Supplementary Figure 3. Electrolyte leakage. Asterisk indicates a significant change in the values calculated by Student's *t*-test (***, $p<0.001$).

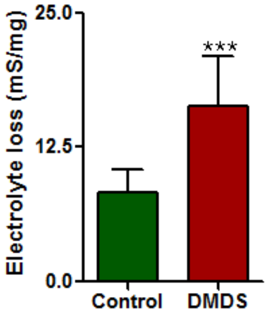

Supplementary Figure 4. Chromatogram showing ergosterol (a) Standard (b) Control (c) DMDS. Ergosterol was determined by HPLC Spectra-Physics equipment with UV 100 detector and a Supelcosil C18 column at room temperature. The mobile phase was HPLC grade methanol: acetonitrile (80:20 v/v). The retention time in these conditions was about 24.5±0.2 min at a flow rate of 0.6 mL/min.

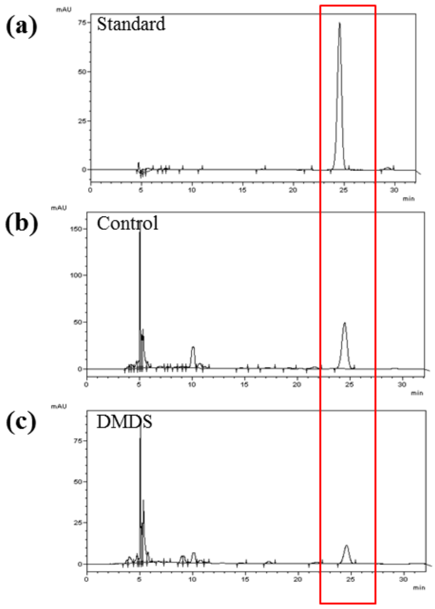

Supplement: Supplementary file 1 — Supplementary Information. [file 41598_2020_63382_MOESM1_ESM.pdf]
